# Metformin improves nonalcoholic fatty liver disease in obese mice via down-regulation of apolipoprotein A5 as part of the AMPK/LXRα signaling pathway

**DOI:** 10.18632/oncotarget.22163

**Published:** 2017-10-30

**Authors:** Min-Jie Lin, Wen Dai, Melanie J. Scott, Rong Li, Yi-Qi Zhang, Yang Yang, Lu-Zhu Chen, Xian-Sheng Huang

**Affiliations:** ^1^ Clinical Skills Training Center, The Second Xiangya Hospital, Central South University, Changsha, Hunan, 410011, China; ^2^ Department of Cardiovascular Medicine, The Second Xiangya Hospital, Central South University, Changsha, Hunan, 410011, China; ^3^ Department of Surgery Labs, University of Pittsburgh, Pittsburgh, PA 15213, USA; ^4^ Department of Stomatology, The Second Xiangya Hospital, Central South University, Changsha, Hunan, 410011, China

**Keywords:** triglyceride, hepatocytes, lipid droplets, leptin-deficient, ob/ob mice

## Abstract

Apolipoprotein A5 (apoA5) has been implicated in the formation of hepatocyte lipid droplets, a histological hallmark of non-alcoholic fatty liver disease (NAFLD). Recent evidence demonstrated that liver X receptor α (LXRα), a transcription factor involved in down-regulation of *APOA5* mRNA, is activated by AMP-activated protein kinase (AMPK) that contributes to metformin-related antihyperglycemic effects. In this study we investigated the role of apoA5 and AMPK/LXRα signaling pathway in metformin-related improvement of NAFLD. Leptin-deficient (*ob/ob*) obese mice with NAFLD were treated with metformin, and signaling pathways were compared with non-metformin treated mice. Additionally, we determined cellular apoA5 and triglyceride (TG) levels in mouse hepatocytes *in vitro* and the effects of metformin, with or without an AMPK inhibitor or LXRα siRNA, on these levels. We found that metformin dose-dependently ameliorated hepatosteatosis and liver dysfunction in *ob/ob* mice, with a significant reduction in hepatic apoA5 expression and TG level. Metformin also dose-dependently increased phosphorylation of hepatic AMPK and LXRα in *ob/ob* mice. Similarly, metformin decreased apoA5 expression and TG level in mouse hepatocytes, with increased phosphorylation of cellular AMPK and LXRα. Addition of AMPK inhibitor or siRNA knockdown of LXRα significantly attenuated metformin-induced down-regulation of cellular apoA5 expression and TG level. AMPK inhibitor also significantly inhibited metformin-induced LXRα phosphorylation in these hepatocytes. Therefore, our findings indicate that metformin improves obesity-related NAFLD via inhibition of hepatic apoA5 synthesis as part of the AMPK/LXRα signaling pathway.

## INTRODUCTION

Non-alcoholic fatty liver disease (NAFLD) represents the most common chronic liver disorder, and is strongly linked to obesity with a reported prevalence as high as 80% in obese population [1, 2]. For obese patients, NAFLD is frequently accompanied by hypertriglyceridemia [3]. NAFLD and hypertriglyceridemia are actually two sides of the same coin, because they both result from disordered triglyceride (TG) metabolism in two interrelated compartments: liver and the plasma [3]. Recent evidence demonstrated that metformin, a first-line antihyperglycemic drug for type 2 diabetes mellitus, effectively improved liver dysfunction in obese patients with NAFLD [4], which indicates that this agent could be enlisted as a promising medicine for obesity-related NAFLD. However, the underlying mechanism of metformin’s effects in NAFLD remain to be elucidated.

Interestingly, accumulated data implicate the potential role of apolipoprotein A5 (apoA5) in the crosstalk between hypertriglyceridemia and NAFLD. Earlier studies implicated apoA5 as a key regulator of plasma TG homeostasis [5, 6], while recent data identified its role in the biogenesis of hepatocyte lipid droplets [7-9], a histological hallmark of NAFLD. More recently, two studies observed overexpression of hepatic *APOA5* mRNA in NAFLD patients, which implicated apoA5 as a potential therapeutic target for NAFLD [10, 11]. *APOA5* mRNA expression is regulated by several factors including liver X receptor α (LXRα), a ligand-activated nuclear transcription factor that has been demonstrated to down-regulate hepatic *APOA5* mRNA expression [12]. Notably, LXRα activation by phosphorylated AMP-activated protein kinase (AMPK), a key factor for pharmacological actions of metformin, has recently been demonstrated to contribute to the antihyperglycemic effects of metformin [13]. Considering the fact that apoA5 is a target gene of LXRα [12], apoA5 could be a potential contributor to metformin-mediated protection against NAFLD. Here, we investigated whether metformin could improve obesity-related NAFLD through inhibition of hepatic apoA5 synthesis regulated via the AMPK/LXRα signaling pathway.

## RESULTS

### Effects of metformin on energy intake, body weight, lipids, apoA5, glucose, insulin, HOMA-IR, and aminotransferases in *ob/ob* mice

As shown in Table 1, compared to controls, *ob/ob* mice in the NAFLD group had significant elevations in energy intake, body weight, lipids, glucose, insulin, the homeostasis model assessment of insulin resistance (HOMA-IR), alanine aminotransferase (ALT), and aspartate aminotransferase (AST). However, metformin effectively decreased these levels in a dose-dependent manner. Interestingly, higher apoA5 levels were observed in NAFLD group than controls, whereas metformin dose-dependently decreased apoA5 levels of these *ob/ob* mice, but these levels were not reduced to control levels.

**Table 1:** Energy intake, body weight, lipids, apoA5, glucose, insulin, HOMA-IR, ALT and AST of mice after four weeks of high fat diet with or without low/high dose metformin

	Control	NAFLD	Low-dose metformin	High-dose metformin
Energy intake (kcal)	13.28 ± 1.59	19.60 ± 2.35^1^	16.82 ± 2.02^1, 2^	15.48 ± 1.85^1, 2, 3^
Body weight (g)	25.12 ± 1.56	57.26 ± 3.07^1^	45.76 ± 2.60^1, 2^	40.05 ± 2.35^1, 2, 3^
Cholesterol (mmol/L)	2.68 ± 0.22	6.05 ± 0.43^1^	4.86 ± 0.35^1, 2^	4.25 ± 0.32^1, 2, 3^
TG (mmol/L)	1.20 ± 0.09	3.12 ± 0.35^1^	2.35 ± 0.26^1, 2^	2.04 ± 0.22^1, 2, 3^
ApoA5 (ng/mL)	112.50 ± 13.48	272.40 ± 29.77^1^	218.05 ± 24.25 ^1, 2^	192.68 ± 20.89 ^1, 2, 3^
Glucose (mmol/L)	6.24 ± 0.33	17.14 ± 0.87^1^	13.79 ± 0.72^1, 2^	10.97 ± 0.55 ^1, 2, 3^
Insulin (mU/L)	10.05 ± 0.42	23.45 ± 1.18^1^	16.88 ± 0.85^1, 2^	13.83 ± 0.70 ^1, 2, 3^
HOMA-IR	2.79 ± 0.13	17.82 ± 0.91^1^	10.35 ± 0.52^1, 2^	6.76 ± 0.34 ^1, 2, 3^
ALT (U/L)	24.52 ± 2.23	162.59 ± 8.12^1^	83.09 ± 3.89 ^1, 2^	71.38 ± 3.62 ^1, 2, 3^
AST (U/L)	20.78 ± 1.89	180.62 ± 9.83^1^	118.63 ± 5.75 ^1, 2^	79.29 ± 4.02 ^1, 2, 3^

^1, 2, 3^ Values were significantly different from control, NAFLD and low-dose metformin group, respectively (*p* < 0.05).

To identify the association of apoA5 with body weight, lipids, HOMA-IR, and liver function (ALT), we performed correlation analyses. After pooling all data of each group, our correlation analyses showed that apoA5 levels were positively correlated with TG (r = 0.58, *p* < 0.01), body weight (r = 0.50, *p* < 0.05), HOMA-IR (r = 0.52, *p* < 0.05), and ALT (r = 0.53, *p* < 0.05), but no significant correlation was found between apoA5 and cholesterol (*p* > 0.05). In addition, a significantly positive correlation was also observed between TG and weight (r = 0.60, *p* < 0.01), body weight and HOMA-IR (r = 0.62, *p* < 0.001), HOMA-IR and ALT (r = 0.56, *p* < 0.05).

### Effects of metformin on hepatic steatosis, TG, apoA5, and phosphorylation of AMPK and LXRα in *ob/ob* mice

**Figure 1:**
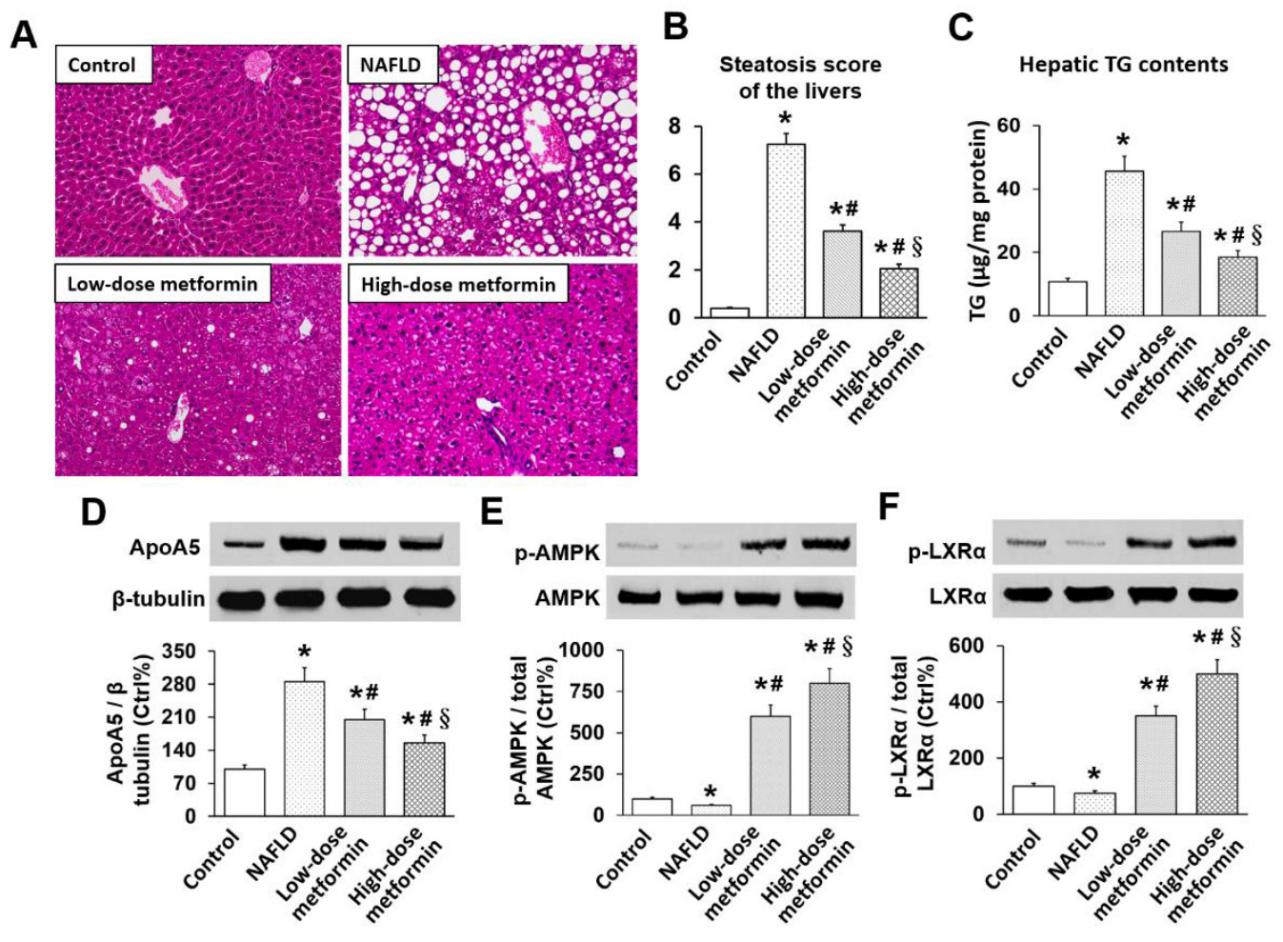
Effects of metformin on hepatic steatosis, TG, apoA5, and phosphorylation of AMPK and LXRα in mice **(A)** H&E histology of liver tissue (×400 magnification); **(B)** Hepatic steatosis score; **(C)** Hepatic TG level; **(D)** Hepatic apoA5 protein levels; **(E)** Hepatic AMPK phosphorylation; **(F)** Hepatic LXRα phosphorylation. ^*^ *p* < 0.05 vs. control; ^#^ *p* < 0.05 vs. NAFLD group; ^§^ *p* < 0.05 vs. low-dose metformin group.

Typical hepatosteatosis, characterized by unstained areas consisting of fat vacuoles on H&E, was observed in livers of NAFLD *ob/ob* mice. Metformin dose-dependently ameliorated hepatosteatosis of these *ob/ob* mice as indicated by a reduction of fat vacuoles (Figure 1A). Steatosis scores in NAFLD mice were significantly higher than controls, and metformin dose-dependently decreased steatosis scores in *ob/ob* mice (all *p* < 0.05) (Figure 1B). Compared with controls, hepatic TG level in NAFLD mice were considerably elevated (10.78 ± 0.98 vs. 45.56 ± 4.76 μg/mg liver protein, *p* < 0.01). Metformin also effectively decreased hepatic TG level in a dose-dependent manner (26.63 ± 2.85 vs. 18.45 ± 2.06 μg/mg liver protein, *p* < 0.05) (Figure 1C). Hepatic apoA5 protein expression in NAFLD mice were significantly elevated compared with control mice, and metformin similarly dose-dependently reduced hepatic apoA5 expressions of these *ob/ob* mice (all *p* < 0.05) (Figure 1D).

We also determined phosphorylation of hepatic AMPK and LXRα in these mice. This was assessed by determining the ratio of phosphorylated (p-AMPK/p-LXRα) to total AMPK/LXRα expression. Although total AMPK expression was not changed, hepatic AMPK phosphorylation of *ob/ob* mice in NAFLD group was lower than controls, and metformin dose-dependently increased AMPK phosphorylation (Figure 1E). Similarly, hepatic LXRα phosphorylation in NAFLD mice was suppressed, while metformin dose-dependently enhanced LXRα phosphorylation of *ob/ob* mice (Figure 1F).

### Effects of metformin on TG, apoA5, and phosphorylation of AMPK and LXRα in hepatocytes

**Figure 2:**
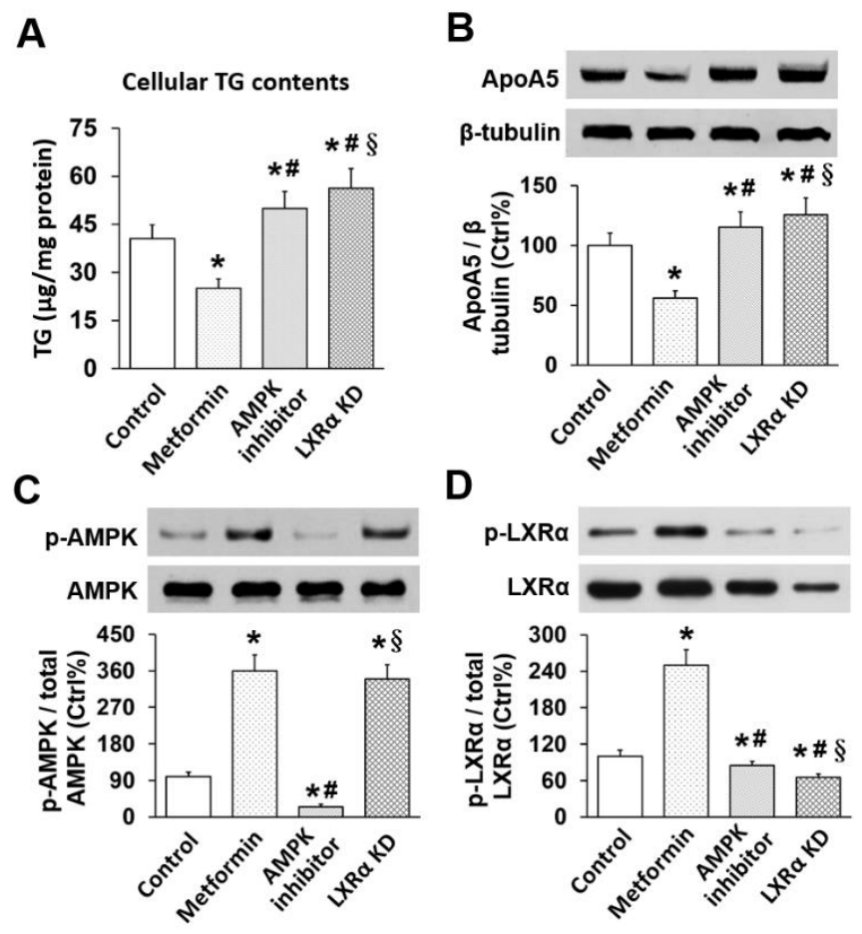
Effects of metformin on TG, apoA5, and phosphorylation of AMPK and LXRα in mouse hepatocytes **(A)** Cellular TG level; **(B)** Cellular apoA5 protein levels; **(C)** Cellular AMPK phosphorylation; **(D)** Cellular LXRα phosphorylation. ^*^ *p* < 0.05 vs. control; ^#^ *p* < 0.05 vs. metformin group; ^§^ *p* < 0.05 vs. AMPK inhibitor group.

Compared to controls, cellular TG level in metformin-treated cells were significantly reduced (40.59 ± 4.25 vs. 25.02 ± 2.98 μg/mg cell protein, *p* < 0.01). However, cellular TG level in AMPK inhibitor-treated and LXRα knockdown (KD) cells were notably increased compared with metformin-treated cells. Cellular TG levels in LXRα KD hepatocytes was higher than in AMPK inhibitor-treated cells (56.29 ± 6.09 vs. 49.95 ± 5.35 μg/mg cell protein, *p* < 0.05) (Figure 2A). Similarly, metformin resulted in a down-regulation of apoA5 expression in hepatocytes, but AMPK inhibitor or LXRα siRNA effectively attenuated metformin-induced reduction of apoA5 expression, and this effect was stronger in LXRα KD cells than in AMPK inhibitor-treated cells (all *p* < 0.05) (Figure 2B).

We also investigated phosphorylation of hepatocyte AMPK and LXRα *in vitro*. As expected, metformin effectively increased AMPK phosphorylation in hepatocytes. When added with the AMPK inhibitor, the metformin-induced phosphorylation of hepatocyte AMPK was significantly repressed, while no inhibitory effect was observed in LXRα KD group (Figure 2C). Of note, LXRα phosphorylation in cells given metformin only was higher than control, which indicated metformin can activate LXRα in hepatocytes. By contrast, AMPK inhibitor and LXRα KD attenuated metformin-induced up-regulation of cell LXRα, and this inhibitory effect was more notable in LXRα KD group (Figure 2D).

## DISCUSSION

In this study we first investigated the effects of metformin on NAFLD in *ob/ob* obese mice, which develop hyperglycemia, insulin resistance (IR), hypertriglyceridemia and fatty livers owing to an inherited deficiency of the appetite-suppressing hormone, leptin [14]. In this study, all *ob/ob* mice developed typical hepatosteatosis, characterized by enriched hepatocyte lipid droplets, hepatic macro- and micro-steatosis, and hypertrophy, suggesting that our obesity-related NAFLD mouse models were successfully established. Interestingly, metformin not only effectively ameliorated hyperglycemia, insulin resistance and hypertriglyceridemia, but also markedly alleviated hepatosteatosis and liver dysfunction in *ob/ob* mice. Therefore, our results demonstrate the beneficial effects of metformin on obesity-related NAFLD, in agreement with previously published data [4, 14]. Consistently with previous pre-clinical [15, 16] and clinical investigations [17, 18, 19], the weight reducing effect of metformin was also revealed in our study. Such weight reduction itself or in combination with other mechanisms of metformin may synergistically contribute to improvement of NAFLD [20]. However, metformin has a modest effect on body weight [21] and the reducing effect of the drug is probably too small to provide a significant hepatic effect [20].

To date, the mechanism underlying the pharmacological actions of metformin on NAFLD remain incompletely understood, but it has been reported that metformin-mediated IR inhibition is partially responsible for its protection against NAFLD [4, 14]. Similarly, our study showed that metformin substantially decreased HOMA-IR (an index of insulin resistance) and plasma ALT levels (an index of liver dysfunction), both being positively correlated with each other, in these *ob/ob* mice. Collectively, these data identified the contribution of metformin-mediated insulin resistance inhibition in amelioration of NAFLD.

A role for apoA5 in metformin-related amelioration of NAFLD was also identified by this study. As mentioned previously, apoA5 is an important player in intrahepatic TG homeostasis, where it promotes the formation of hepatocyte lipid droplets [7-9], a histological hallmark of NAFLD. Similarly, simultaneously elevated TG contents and apoA5 expression within the livers were detected in our *ob/ob* mice with NAFLD. Together with our previous findings that APOA5 KD resulted in a significant decrease of hepatocyte TG level [22], our data demonstrate the crucial role of apoA5 in intrahepatic TG accumulation. Pathologically, excessive intrahepatic TG accumulation inevitably leads to NAFLD [23], indicating the potential role of apoA5 in the pathogenesis of NAFLD, which has recently also been identified by two independent research groups [10, 11]. Ress *et al* observed an elevation of hepatic APOA5 mRNA in obese patients with NAFLD, which were markedly down-regulated after improvement in hepatosteatosis [10]. Similarly, Feng *et al* detected increased expression of APOA5 mRNA in pediatric NAFLD livers that was positively associated with increased hepatic TG level. They furthermore found an increase of hepatic APOA5 mRNA in obese rats with NAFLD [11]. Similarly, our study demonstrated that obese mice with NAFLD had higher hepatic apoA5 expression and TG level, and metformin treatment resulted in a considerable reduction of both hepatic apoA5 expression and TG level in livers of these animals. Moreover, metformin simultaneously decreased apoA5 expression and TG level in mouse hepatocytes *in vitro*. Together with our findings that metformin effectively ameliorated hepatosteatosis of these obese mice, we conclude that metformin protection against NAFLD is associated with its inhibition of hepatic apoA5 production.

We also made another interesting finding about plasma apoA5 and TG in this study. Plasma apoA5 levels were increased in *ob/ob* mice despite their hypertriglyceridemic state, and metformin simultaneously decreased plasma apoA5 and TG levels. Additionally, a positive correlation between the two factors was identified in this study. These findings are consistent with data obtained in our previous study, and a “response-to-hypertriglyceridemia” hypothesis was proposed that plasma apoA5 elevation could be an adaptive consequence of hypertriglyceridemia and *vice versa* in these *ob/ob* mice after treatment with metformin [22]. Similar phenomena were observed in other hypertriglyceridemia-related diseases, including type 2 diabetes [24], severe hypertriglyceridemia [25], and acute coronary syndrome [26], where plasma apoA5 levels were elevated and positively correlated with TG.

Given that metformin-associated pharmacological actions are mainly dependent on the AMPK signaling pathway [27], we investigated the importance of AMPK in metformin-induced down-regulation of apoA5. As expected, metformin treatment led to AMPK phosphorylation in animals and hepatocytes, which was accompanied by a significant reduction of apoA5 expression and TG level. However, administration of a selective AMPK inhibitor (compound C) almost eliminated the inhibitory effects of metformin on hepatocyte apoA5 expressions and TG level. Thus, our findings indicate that AMPK activation is required for metformin-mediated inhibition of hepatic apoA5 expression and TG production, which is believed to prevent NAFLD development.

Considering the documented role of LXRα in regulation of hepatic APOA5 mRNA [12], we further investigated the involvement of LXRα in metformin-induced apoA5 down-regulation. Jakel *et al* found that LXRα activation resulted in down-regulation of hepatic APOA5 mRNA [12]. By contrast, our study demonstrated that LXRα KD effectively eliminated the inhibitory effects of metformin on hepatocyte apoA5 expression. Actually, the AMPK/LXRα signaling pathway was recently confirmed as important in the antihyperglycemic effects of metformin [13]. In this current study, we observed that addition of the AMPK inhibitor pronouncedly repressed hepatocyte LXRα phosphorylation by metformin. Therefore, this study also implicates the AMPK/LXRα signaling pathway in metformin-induced inhibition of hepatic apoA5 synthesis.

In summary, our findings implicate apoA5 in metformin-mediated amelioration of obesity-induced NAFLD. Metformin down-regulates hepatic apoA5 expression and reductes hepatic TG level, which contributes to prevention of NAFLD development, and involves the AMPK/LXRα signaling pathway. Nevertheless, the clinical significance of our findings, and in particular apoA5 as a potential target for NAFLD management, remains to be investigated in future clinical trials.

## MATERIALS AND METHODS

### Animal study

Experiments were carried out in accordance with a protocol approved by the Institutional Animal Care and Use Committee of Animal Experiment Department of Central South University. Adequate measures were taken to minimize pain or discomfort to experimental animals. Mouse NAFLD model was established as previously described [28]. Animals used in this study were 5-week-old male wild-type (WT) and *ob/ob* mice on a C57BL/6J genetic background (Animal Center of Nanjing University, Nanjing, China). Animals were housed in specific pathogen-free conditions at constant temperature (21 ± 1°C) and humidity (50 ± 5%) under 12-hour light/dark cycle. All mice received a standard laboratory chow (10% of calories derived from fat, 3.85|kcal/g) and water available *ad libitum*. The results of the food intake were obtained from the mice individually housed in cages; the body weight of the freely available chow in a jar was measured every 3 days, and the daily food intake was calculated. Metformin was dissolved in drinking water and administered by daily oral gavage. After 1 week of adaptation, *ob/ob* mice were randomized into 3 groups, including NAFLD, low- and high-dose metformin groups, with WT mice as controls (n = 10 each group). The mice in low- and high-dose metformin groups were treated with metformin 50 and 100 mg/kg, respectively. After 4 weeks, animals were fasted overnight and sacrificed under anesthesia with sodium pentobarbital. Blood samples were collected in 5% EDTA tubes stored at −80°C for measurements. Animal livers were excised, with part fixed in 10% neutral formalin solution, and the remainder frozen immediately in liquid nitrogen and stored at −80°C until use. Measurements of plasma apoA5, lipids, glucose, insulin, ALT, AST, HOMA-IR, and hepatic TG contents were conducted as described previously [13].

### Histological analysis of mouse hepatic tissue

Liver tissues were fixed, dehydrated and embedded in paraffin. Paraffinized tissue sections (4 μm thickness) were stained with hematoxylin and eosin (H&E) for microscopic evaluation of the degree of NAFLD. Steatosis score is defined by the sum of scores of macrosteatosis, microstetosis, and hypertrophy [29], with each given a score of 0 to 3 assessed from three randomly selected fields of each liver section. The sum of these scores (steatosis score) was calculated for each fields. The mean score for three fields was calculated as the steatosis score for the specimen, and liver histology of 6 mice per group was examined.

### Hepatocyte experiment *in vitro*

Murine hepatic IAR20 cells were cultured as previously described [13]. All chemicals were dissolved in DMSO and the final concentration of DMSO in media was maintained at 0.1% (v/v). When the cells reached ∼50% confluence, they were cultured in serum-free media and divided into 4 groups: (1) control group, treated with the scrambled control siRNA; (2) metformin group, with 500 μM metformin; (3) AMPK inhibitor group, with 500 μM metformin and 40 μM compound C, a selective inhibitor of AMPK (Abcam); (4) LXRα KD group, transfected with LXRα-targeting siRNA and then treated with 500 μM metformin. At collection, all cells were washed and cellular TG was measured by enzymatic reagents [13].

### Transient cell transfection with siRNAs

As described previously [13], IAR20 cells were transiently transfected with siRNA targeting LXRα (100 nM) or control, scrambled siRNA (Santa Cruz Biotechnology, California, USA) using Lipofectamine 2000 (Invitrogen, California, USA). The sequences used for LXRα KD were sense 5′-CGU AGC AUU AAG GGA GAG U-3′ and antisense 5′-ACU CUC CCU UAA UGC UAC G-3′. The sequences for the control siRNA were sense 5′-CCU ACG CCA CCA AUU UCG U-3′ and antisense 5′-ACG AAA UUG GUG GCG UAG G-3′. The transfected cells were stabilized for 48 hours before subsequent treatment(s).

### Western blot analysis

As described previously [13], equal protein amounts of whole cell/tissue lysates were loaded into lanes and separated on 10% SDS-PAGE and transferred onto PVDF membranes. The membranes were then blocked with 5% nonfat milk in Tris-buffered saline containing 0.05% Tween 20 (TTBS) at room temperature for 1 hour and incubated at 4°C for overnight with a primary antibody diluted 1:1000 to 1:5000 in 5% nonfat milk in TTBS. The primary antibodies used were against apoA5 (Abcam, Cambridge, USA), AMPK and p-AMPK (Cell Signaling, Massachusetts, USA), LXRα and p-LXRα (Abcam, Cambridge, USA). After incubation with a HRP-conjugated secondary antibody (1:5000), immunoreactive bands were visualized using an Enhanced Chemiluminescence Plus Kit (GE Healthcare, Pittsburgh, USA), followed by exposure to X-ray film.

### Statistical analysis

All statistical calculations were performed with SPSS 15.0 statistical software package (SPSS). Data are presented as mean ± SD. When two groups were compared, the significance was evaluated by unpaired Student t-test; when multiple groups were compared, the significance was evaluated by one-way ANOVA followed by the test of Student-Newman-Keuls. Coefficients of correlation (r) were calculated by the Pearson correlation analysis. Results were considered statistically significant at two-sided *p* < 0.05.

## Abbreviations

ApoA5: apolipoprotein A5
NAFLD: non-alcoholic fatty liver disease
LXRα: liver X receptor α
AMPK: AMP-activated protein kinase
TG: triglyceride
ALT: alanine aminotransferase
AST: aspartate aminotransferase
HOMA-IR: homeostasis model assessment of insulin resistance
H&E: hematoxylin and eosin
KD: knockdown
TTBS: Tris-buffered saline containing 0.05% Tween 20
